# Slimming using magistral formulas: what are the risks?

**DOI:** 10.1080/07853890.2021.1896111

**Published:** 2021-09-28

**Authors:** Dulce Laúdo, Tânia Fernandes, Ana I. Fernandes

**Affiliations:** ^a^PharmSci Lab, Centro de Investigação Interdisciplinar Egas Moniz (CiiEM), Egas Moniz Cooperativa de Ensino Superior, Caparica, Portugal

## Abstract

**Introduction:**

Magistral formulas (MF) are prepared by the pharmacist for a given patient according to a prescription and following technical and scientific compounding standards. MF are often used in weight loss regimens and contain blends of drugs (D) and plant (P) extracts. Associations potentiate interactions and related adverse effects, compromising effectiveness and risking the patient’s health [1,2]. Thus, the purpose of this work is to give an overview of MF intended for slimming, prescribed by doctors, in a perspective of efficacy and safety.

**Materials and methods:**

Slimming MF (prescribed to overweight women, as hard gelatine capsules, once or twice daily) were obtained in pharmacies and analysed in terms of labelled drug/bioactive composition and dosage, therapeutic indication/claim, recommended daily dose (RDD), side effects/interactions and contraindications. Written consent for data use was obtained.

**Results:**

MF did not contain unlawful ingredients [3]. Actives were used mostly in sub therapeutic doses (Table 1). Weight loss is a result of (a) side effect of D-III/IV (off-label use), (b) water loss due to therapeutic action (D-I/IX and P-V/VII/VIII), or (c) claimed appetite reduction (P-VI/X/XI).

**Discussion and conclusions:**

Off-label uses of drugs and efficacy of sub therapeutic doses are questionable. D-II,III present risk of abuse and dependence. Combination of laxatives (MF 1, 2 and 6) is not recommended, increasing the chances of electrolyte imbalance and dehydration, and reducing absorption of ansa diuretics. Clinical data to support the claims and posology of botanicals is scarce and contradictory; moreover, potential side effects/interactions are at times unknown and adulteration/contamination is a risk. Of note is the potential for interaction of P-V (inhibiting several isoenzymes of CYP450) and the association of D-I/P-V may cause hypovolemia and hypocaliemia. Even if no additional interactions were found between molecules, combinations may increase the risk of adverse events. Severe/fatal interactions may occur with other drugs (e.g. D-II + opioids; D-III + MAO inhibitors), so knowledge of patient's clinical history and related medication is of the utmost importance, when prescribing and counselling. Indeed, evaluation of safety and efficacy of MF is a shared responsibility of doctor and pharmacist and requires robust scientific data, especially regarding botanicals. Slimming medication alone, without lifestyle changes, is not effective in the long term and may pose a health risk, as pointed out in this study.

**Table 1.** MF labels composition and dose (mg).

Active ingredient (AI)	MF1	MF2	MF3	MF4	MF5	MF6	MF7	Main therapeutic indication / claim	Usual posology in major pathology
Furosemide^I^	20	25		18	18	30		Diuretic	20–80/120 mg/day
Chlordiazepoxide^II^	8	8		8	8	10		Anxiolytic	30 mg (3 times/day)
Bupropion^III^	120	100		140	150	130		Antidepressant	150 mg (2 times/day)
Metformine^IV^	280		250	300	300	260		Antidiabetic	500 mg (2/3 times/day)
Artichoke^V^	110	400						Laxative	500 mg/day*
Bitter orange *(Citrus aurantium)*^VI^	150			200	200	200	200	Appetite reducer	50–100 mg/day*
*Centella asiatica* L.^VII^		400	400				750	Anti-cellulite, venotonic	60–120 mg/day*
Cascara Sagrada (*R. purshiana*)^VIII^		100		130		120		Laxative	150–325 mg/day*
Phenolphthalein^IX^	65	100			90	85		Laxative	30–200 mg/day
Glucomannan (*A. konjac*)^X^							500	Appetite reducer	1000–13,000 mg/day*
Slimalluma *(Caralluma fimbriata)*^XI^		300	250					Appetite reducer	500 mg (2 times/day)*

Roman superscripts identify the substance in the text.

*RDD not well established.
